# *Burkholderia pseudomallei* in Sarawak, Malaysian Borneo, Remains Highly Susceptible to Trimethoprim-Sulfamethoxazole Despite Resistance to Its Individual Components

**DOI:** 10.3390/pathogens15010110

**Published:** 2026-01-19

**Authors:** Liana Lantong Sumbu, Tonnii Loong-Loong Sia, Mong-How Ooi, Anand Mohan, Jin-Shyan Wong, Yuwana Podin

**Affiliations:** 1 Institute of Health and Community Medicine, Universiti Malaysia Sarawak, Kota Samarahan 94300, Sarawak, Malaysia; klsliana94@gmail.com (L.L.S.); monghowooi@gmail.com (M.-H.O.); anand_bintulu@yahoo.com (A.M.); 2 Department of Medicine, Miri Hospital, Miri 98000, Sarawak, Malaysia; tonnii_sia@moh.gov.my; 3 Department of Paediatrics, Sarawak General Hospital, Kuching 93586, Sarawak, Malaysia; 4 Department of Paediatrics, Bintulu Hospital, Bintulu 97000, Sarawak, Malaysia; 5 Department of Internal Medicine, Borneo Medical Centre, Kuching 93350, Sarawak, Malaysia; gangrenes@yahoo.com

**Keywords:** melioidosis, *Burkholderia pseudomallei*, gentamicin-susceptible, Sarawak, antibiotic susceptibility, trimethoprim-sulfamethoxazole

## Abstract

*Burkholderia pseudomallei*, the causative agent of melioidosis, is endemic in Sarawak, Malaysian Borneo, where it is represented by a unique gentamicin-susceptible population. Despite trimethoprim-sulfamethoxazole (co-trimoxazole) being the cornerstone of eradication therapy, emerging reports of elevated minimum inhibitory concentrations (MICs) among Sarawak isolates have raised concerns over its clinical efficacy. We performed a retrospective and comprehensive antibiotic susceptibility assessment of clinical *B. pseudomallei* isolates from hospitals across Sarawak. Susceptibility to trimethoprim-sulfamethoxazole was determined using disk diffusion and the E-test, interpreted by both CLSI and EUCAST guidelines. Resistance to the individual components, trimethoprim and sulfamethoxazole, was characterized by broth microdilution. The results demonstrated a high prevalence of trimethoprim-sulfamethoxazole susceptibility, with 96.3% of isolates susceptible by CLSI criteria and 97.6% by EUCAST criteria. Interestingly, broth microdilution revealed that resistance to trimethoprim and sulfamethoxazole individually did not confer resistance to the synergistic combination. Our analysis validated CLSI guidelines as the most reliable standard for antimicrobial resistance surveillance in this region. This study provides evidence that trimethoprim-sulfamethoxazole remains effective for melioidosis treatment in Sarawak, offering crucial reassurance to clinicians. The paradoxical finding of susceptibility to the drug combination despite resistance to its individual components underscores the critical importance of the synergistic activity of trimethoprim-sulfamethoxazole and highlights the need for further investigation into the molecular basis of resistance in this distinct *B. pseudomallei* population.

## 1. Introduction

Melioidosis is a significant cause of community-acquired sepsis and pneumonia in its area of endemicity [1]. While historically endemic in Northern Australia and parts of Southeast Asia, its prevalence is rising with emerging foci in the Americas and Africa [2,3,4]. Recent models project an annual global burden of 165,000 affected individuals and 89,000 deaths [2]. In Malaysia, melioidosis is responsible for approximately 3700 cases and more than 2000 deaths yearly [5]. While Sarawak, the largest state in Malaysia, represents a significant hotspot, with the central regions reporting an average incidence of 12.3 per 100,000 and a case-fatality rate of up to 35% [6,7].

The causative agent of melioidosis is *Burkholderia pseudomallei*, a Gram-negative saprophyte classified as a Tier 1 select agent by the US Centers for Disease Control and Prevention [8]. This designation reflects its potential of easy dissemination, high fatality rate, intrinsic resistance to myriads of antibiotics, and lack of a licensed vaccine [2].

Standard melioidosis treatment employs biphasic therapy, with the initial parenteral intensive-phase of β-lactams such as ceftazidime or carbapenem, followed by the eradication-phase of oral trimethoprim-sulfamethoxazole (co-trimoxazole) regimen [9]. However, concern has grown recently regarding the efficacy of the trimethoprim-sulfamethoxazole regimen in Sarawak, following the emerging reports of an elevated minimal inhibitory concentration (MIC) of the clinical *B. pseudomallei* isolates [10,11]. This concern is particularly significant since the Sarawak isolates are predominantly characterized by a unique gentamicin-susceptible genotype unreported elsewhere [12], suggesting a distinct evolutionary lineage that may exhibit an atypical resistance profile. Additionally, there is a lack of standardized interpretive criteria for susceptibility testing for the Sarawak isolates that often complicates clinical reporting and optimal treatment guidance.

To address these critical gaps, this study aimed to evaluate the prevalence of trimethoprim-sulfamethoxazole resistance and to understand the mechanism of such phenomena in Sarawak’s distinct *B. pseudomallei* clinical isolates. We performed a retrospective analysis using recommended antibiotic susceptibility testing methods such as disk diffusion, the E-test, and broth microdilution for trimethoprim-sulfamethoxazole and its individual components. The study findings highlight crucial susceptibility data of the Sarawak isolates and reaffirm the clinical utility of trimethoprim-sulfamethoxazole for melioidosis management in Sarawak. The study also identified isolates with divergent susceptibility to the individual components, a finding that warrants further molecular characterization to elucidate the underlying genetic mechanism.

## 2. Materials and Methods

### 2.1. Ethics Statement

This study was approved by the Medical Research Ethics Committee and registered with the National Medical Research Registrar (NMRR-16-1029-31390) and the Clinical Research Centre, Ministry of Health Malaysia. Anonymized bacterial isolates were obtained from archival collections and routine laboratory diagnostic procedures; hence, the requirement for written patient consent was waived.

### 2.2. Bacterial Strains

A total 164 clinical *B. pseudomallei* isolates were included in this study, collected from melioidosis cases reported between 2011 and 2018 in six hospitals located in Bintulu, Sibu, Kapit, Miri, and Kuching in Sarawak, Malaysian Borneo (Table 1). The prototype strain SWK-C 001 (also coded as MSHR5078) was used for antibiotic susceptibility testing (AST) optimization. *Escherichia coli* ATCC 25922 and *Escherichia coli* ATCC 11775 were used as the quality control (QC) in the broth microdilution assays. All strains were cultured on modified Ashdown’s selective agar and incubated at 37 °C for 24 h. The medium contained 50 µg/mL colistin in place of gentamicin, based on previous findings [13]. Tryptic soy agar (TSA) (OXOID, Basingstoke, Hamsphire, UK) was used as an intermediary medium for all pure cultures prior to preparation for antimicrobial susceptibility testing and broth microdilution assays.

### 2.3. Antibiotic Susceptibility Testing (AST)

**Figure 1:**
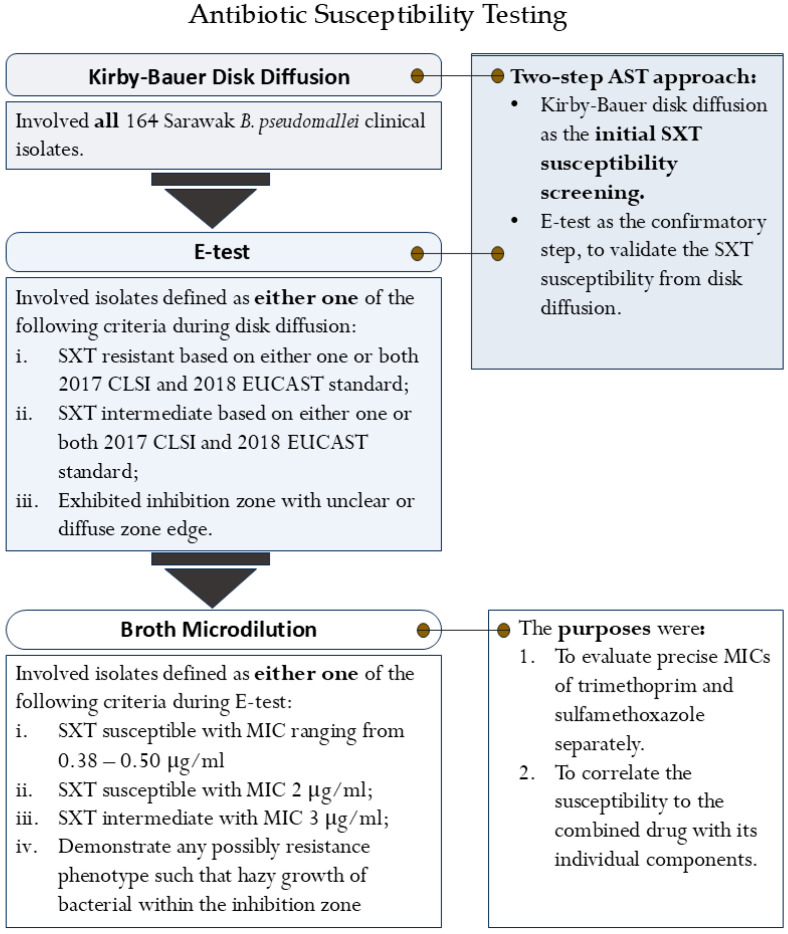
The flowchart of the three standard antibiotic susceptibility testing procedures.

The trimethoprim-sulfamethoxazole susceptibility of the Sarawak *B. pseudomallei* clinical isolates was evaluated using a tiered testing strategy, performed in accordance with Clinical and Laboratory Standard Institute (CLSI) and European Committee on Susceptibility Testing (EUCAST) guidelines [14,15,16]. Initial screening was conducted with Kirby–Bauer disk diffusion, the routine method in most hospital settings in Sarawak. Selected isolates from this screen were then subjected to confirmatory E-testing. Finally, broth microdilution was employed to determine precise MICs for both sulfamethoxazole and trimethoprim separately. This final procedure was outlined to correlate the susceptibility to the combined drug with its individual components. The specific selection criteria of bacterial isolates for each procedure are detailed in Figure 1.

#### 2.3.1. Kirby–Bauer Disk Diffusion Testing

The Kirby–Bauer disk diffusion method was performed according to the updated and modified protocols regulated by the 2017 CLSI guideline [14]. Bacterial suspensions were adjusted to a 0.5 McFarland standard and lawn-cultured onto Mueller-Hinton agar (MH) (HiMedia Laboratories, Mumbai, India). A 25 μg trimethoprim-sulfamethoxazole disk (OXOID, Basingstoke, Hamsphire, UK) was applied onto the agar plate, followed by incubation at 37 °C for 18 h. Inhibition zone diameters for susceptible, intermediate, and resistant according to CLSI were defined as ≥16 mm, 11–15 mm, and ≤10 mm; and for the EUCAST standard, they were ≥14 mm, 11–14 mm, and <11 mm, respectively [14,15].

#### 2.3.2. E-Test

MICs for selected isolates against trimethoprim-sulfamethoxazole were determined using E-test strips (bioMérieux, Marcy-I'Étoile, France) according to the manufacturer’s instructions. Similarly, strains were grown overnight on TSA before the MIC determination. For isolates that were selected for broth microdilution (Figure 1), susceptibility to a panel of clinically relevant antibiotics (amoxicillin-clavulanate, ceftazidime, doxycycline, and meropenem) was further evaluated to assess their multidrug-resistant profiles. Gentamicin and azithromycin susceptibility was employed to determine the antibiogram phenotype of the isolates. The interpretation of MICs was according to CLSI and EUCAST standards for *B. pseudomallei* [14,15], with azithromycin interpreted using the manufacturer’s recommendation for aerobes (Table 2), as no standard breakpoints were established for the *non-Enterobacteriaceae*.

#### 2.3.3. Broth Microdilution

The susceptibility of the selected isolates to trimethoprim (TMP) and sulfamethoxazole (SMX) was determined separately using the broth microdilution procedure outlined in CLSI documents M07-A11 and M100 [14,16]. Tests were performed using Mueller Hinton II broth (HiMedia Laboratories, Mumbai, India) at pH 7.0, using a final inoculum of 5 × 10^5^ CFU/mL, and incubated at 37 °C for 18 h. The inoculum was tested against antibiotic concentrations ranging from 0.25 µg/mL to 128 µg/mL for trimethoprim and 2 µg/mL to 1024 µg/mL for sulfamethoxazole. All tests were performed in a minimum of biological triplicate. The final adjusted optical density (OD) value was calculated using Equation (1), and the epidemiological MIC values were derived from Equation (2). Although growth inhibition was analyzed at both 50% (MIC_50_) and 80% (MIC_80_) thresholds, the MIC_80_ was used for reporting. MIC_80_ breakpoints interpretation for susceptible and resistant was defined as ≤8 µg/mL and ≥16 µg/mL and ≤256 µg/mL and ≥512 µg/mL for trimethoprim and sulfamethoxazole, respectively.

(1) $$\mathrm{Final}\;\mathrm{OD}\;\mathrm{value}=\frac{\left(\mathrm{OD}\;\mathrm{value}\;1+\mathrm{OD}\;\mathrm{value}\;2+\mathrm{OD}\;\mathrm{value}\;3\right)}{3}$$

(2) $$\mathrm{Bacterial}\;\mathrm{kill}\;\%=\frac{(\mathrm{Growth}\;\mathrm{control}\;\mathrm{OD}\;\mathrm{value}-\mathrm{Final}\;\mathrm{OD}\;\mathrm{value})}{\mathrm{Growth}\;\mathrm{control}\;\mathrm{OD}\;\mathrm{value}}\;\times \;100\%\;$$

Quality Control (QC)

*E. coli* ATCC 25922 was initially employed as the primary QC strain. However, due to inconsistent and deranged MICs, *E. coli* ATCC 11775 was employed as an alternative QC strain. Since it is not standardized for AST, optimization and validation of its MICs against trimethoprim and sulfamethoxazole were carried out in triplicate against those of *E. coli* ATCC 25922 and a panel of *B. pseudomallei* with known susceptibility profiles. The alternative QC strain, *E. coli* ATCC 11775 demonstrated consistent MIC values within the expected ranges for *B. pseudomallei* and proved stable even with prolonged incubation (36 h), confirming its reliability for this assay (Table 3 and Table 4).

### 2.4. Statistical Data Analysis

All MIC definitions from the three antibiotic susceptibility testing methods were analyzed using SPSS Statistics version 23.0 (IBM, USA). The agreement between CLSI and EUCAST interpretive guidelines was assessed using the Cohen Kappa (κ) coefficient. The resulting Cohen’s Kappa value (κ) was interpreted as follows [17]:

κ **_=_** 0.00–0.20 indicates slight agreement
κ **_=_** 0.21–0.40 indicates fair agreement
κ **_=_** 0.41–0.60 indicates moderate agreement
κ **_=_** 0.61–0.80 indicates substantial agreement
κ **_=_** 0.81–1.00 indicates almost perfect agreement

Subsequently, trimethoprim-sulfamethoxazole susceptibility was analyzed using descriptive statistics and a corrective mathematical estimation. First, the results from the initial disk diffusion testing were summarized descriptively. Next, a corrected susceptibility frequency was calculated by comparing these results against those from the confirmatory E-test. This involved identifying all isolates with susceptibility verified by the E-test. The final, corrected overall frequency was then derived by adjusting the initial disk diffusion data to reclassify isolates initially misclassified as resistant or intermediate.

### 2.5. Genomic Analysis

#### 2.5.1. Primer Design

Oligonucleotide primers (Table 5) targeting the *bpeEF-oprC* efflux pump genes cluster (*bpeT-llpE-bpeE-bpeF-oprC*) were designed using Primer3plus version 2.6.1 [18], with *B. pseudomallei* K96243 (accession number: BX571996.1) as the reference strain [19]. This gene cluster has previously been associated with the trimethoprim-sulfamethoxazole resistance mechanism [20,21].

#### 2.5.2. Polymerase Chain Reaction (PCR)

Genomic DNA was extracted using Chelex^®^ 100 Resin (Bio-Rad, Hercules, CA, USA). The PCR was performed in a 15 μL reaction volume containing 1× Taq Polymerase Buffer, 0.2 mM dNTPs, 0.08 µ/μL Taq Polymerase, primers, and 1 μL of a DNA template. Amplification parameters were set at 95 °C for 5 min of initial denaturation, 45 cycles of 95 °C for 30 s, 52–55 °C for 30 s, and 72 °C for 1 min followed by a final extension at 72 °C for 5 min [22].

For PCR product visualization, 5 μL of the PCR products were mixed with 1 μL of 6× loading dye and electrophoresed on a 0.7% agarose gel in 1× TBE buffer alongside a 100 bp DNA ladder (Vivantis, Shah Alam, Malaysia). Gels were run at 70 V, stained with SYBR^®^ Safe DNA gel stain (Invitrogen, Carlsbad, CA, USA), and visualized using a Gel Doc XR+ System (Bio-Rad, Hercules, CA, USA).

## 3. Results

To accurately evaluate the trimethoprim-sulfamethoxazole susceptibility and to address the concerns for false-resistant results from disk diffusion, a two-step AST approach was implemented. Following the initial screening by disk diffusion, E-tests were used to validate the susceptibility results. The inhibition zone (IZ) and MIC from both methods were interpreted in parallel using CLSI and the EUCAST guidelines [14,15]. This approach allowed for the analysis of the interpretation variability between standards to determine the valid interpretive standard for *B. pseudomallei* in Sarawak.

### 3.1. Disk Diffusion Testing Revealed a High Prevalence of Trimethoprim-Sulfamethoxazole Susceptibility Among the Sarawak B. pseudomallei

**Figure 2:**
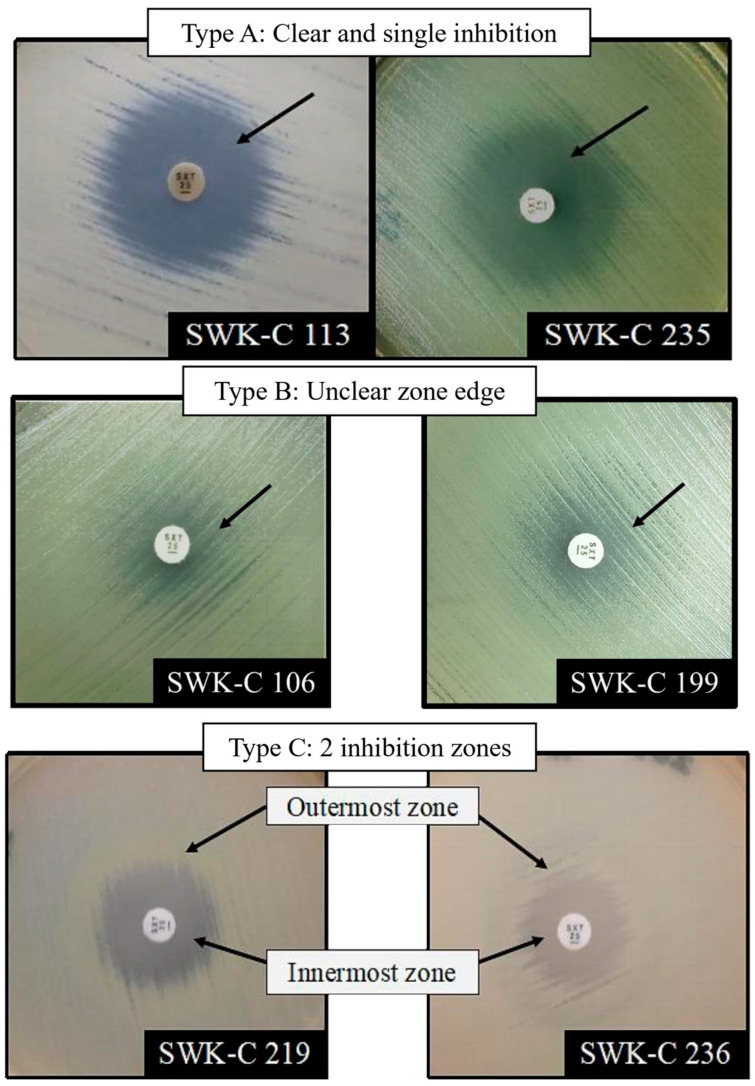
Type of inhibition zone(s) formed by Sarawak clinical *B. pseudomallei* isolates.

Of 164 isolates tested against a 25 μg trimethoprim-sulfamethoxazole disk (Oxoid, Basingstoke, Hamsphire, UK), 117 exhibited a single IZ, while the remaining 47 formed double IZs. Our assessment identified three distinct phenotypic IZ patterns (Figure 2). Isolates with a single IZ typically exhibited Type A or Type B patterns. Type A is a clear inhibition zone, while Type B was characterized by an unclear or diffuse edge that complicated IZ diameter measurement. All isolates with double IZs displayed the Type C pattern, characterized by an inner zone where 80% of growth was inhibited (MIC_80_) and an outer zone of 50% inhibition (MIC_50_). Geographically (see Appendix A Table A1), isolates from Bintulu and Kapit showed a notable tendency to form Type B and C IZs, which complicate the susceptibility definition. Most isolates exhibiting the diffused Type B pattern originated from Bintulu (21 isolates) and Kapit (5 isolates). Additionally, 8 isolates from these regions exhibited Type C IZ. The isolates were recorded with intermediate and resistant diameters ranging from 12 to 15 mm for Bintulu isolates and 9 to 15 mm for Kapit isolates.

For all isolates, trimethoprim-sulfamethoxazole susceptibility was determined by measuring the IZ diameter at the MIC_80_ zone edge (see Table 6). The modal IZ diameter was 20 mm, observed in 25/164 isolates. The majority of the isolates (134/164) exhibited a zone diameter greater than 15 mm, while 30/164 isolates showed a diameter below this threshold. Regardless of whether single or double IZs formed, the distribution showed that most Sarawak isolates in this collection were susceptible to trimethoprim-sulfamethoxazole.

The analysis highlights the differences between the two guidelines (see Table 7). According to the CLSI guidelines, the results translated to 81.7% susceptibility and 18.3% reduced susceptibility (including intermediate and resistant isolates). In contrast, EUCAST criteria classified 131/164 isolates (79.9%) as susceptible and 33/164 (20.1%) as non-susceptible. The interpretative discrepancy was reflected in a Cohen’s Kappa value of κ = 0.458 (95% CI: 0.340–0.570), indicating only moderate agreement between the two guidelines. This observation was largely attributable to isolates with challenging IZ phenotypes (Type B in Figure 2) and compounded by the different interpretations by the two guidelines. CLSI criteria define the zone margin by disregarding slight (≤20%) inner-zone growth, whereas EUCAST advises ignoring minor growth only if a clear zone edge is visible. Therefore, while Sarawak isolates demonstrated high trimethoprim-sulfamethoxazole susceptibility, their susceptibility definition was dependent on both the observed IZ phenotype and the interpretive standard applied.

### 3.2. E-Test Indicates Evidence of a Trimethoprim-Sulfamethoxazole False-Resistance Profile Among the Sarawak B. pseudomallei Clinical Isolates

**Figure 3:**
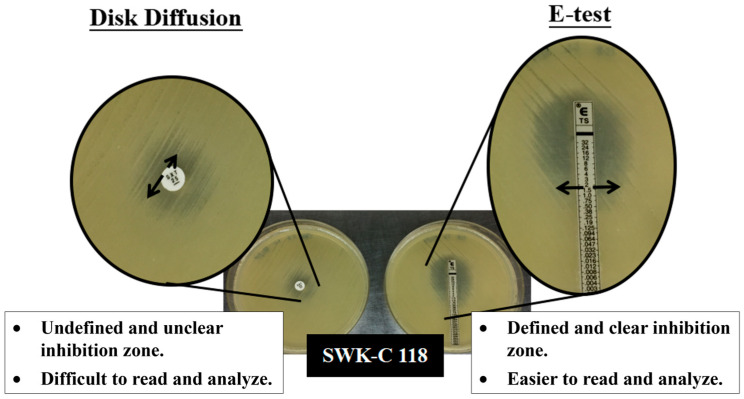
Inhibition zones of Sarawak *B. pseudomallei* clinical isolates by disk diffusion and E-test.

To validate the trimethoprim-sulfamethoxazole susceptibility by disk diffusion, the E-test was performed on 42 isolates previously defined as either intermediately susceptible or resistant (see bolded isolates in Appendix A Table A1). This confirmation was essential due to the observed challenges in interpreting IZs with hazy growth or unclear margins from the disk diffusion method (Figure 3).

The E-test yielded clear and easily interpretable IZs for all isolates. The resulting MIC values indicated that the majority of these isolates were susceptible. The MICs for susceptible isolates ranged from 0.38 to 2 μg/mL, with a predominant MIC of 0.75 μg/mL (see Appendix A Table A2). Only one isolate, SWK-C 118, exhibited an MIC of 3 μg/mL, classifying it as intermediate.

Based on these definitive E-test results, the overall trimethoprim-sulfamethoxazole susceptibility prevalence for the studied Sarawak *B. pseudomallei* isolates was mathematically corrected. The estimated susceptibility was 96.3% (158/164) by CLSI standards and 97.6% (160/164) by EUCAST standards (Table 8).

### 3.3. Broth Microdilution Demonstrated the Resistance of Sarawak B. pseudomallei Clinical Isolates to Trimethoprim and Sulfamethoxazole Despite Trimethoprim-Sulfamethoxazole Susceptibility

To further evaluate the susceptibility patterns observed in disk diffusion and the E-test, a subset of 14 isolates was selected for broth microdilution analysis. This selection included isolates with intermediate MIC values (i.e., 3 μg/mL), atypical inhibition zones such as Type B and C in Figure 2, and those representing comparatively low MIC values within the range of 0.38 to 0.50 μg/mL.

The initial characterization (Table 9) of this subset confirmed that 12/14 (85.7%) isolates were azithromycin- and gentamicin-susceptible (Gen^S^), consistent with the established predominant and unique genotype of the *B. pseudomallei* isolates from Sarawak [12,13]. All 14 isolates were susceptible to the clinically relevant melioidosis antibiotics, including meropenem, ceftazidime, doxycycline, and amoxicillin-clavulanate. Gen^S^ isolates exhibited higher MIC values ranging from 0.50 μg/mL to 2 μg/mL, whereas the MIC values of the gentamicin-resistant (Gen^R^) isolates ranged from 0.38 to 0.50 μg/mL.

**Figure 4:**
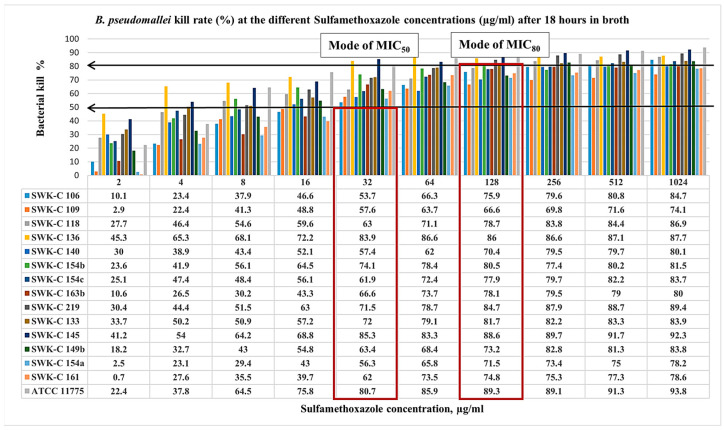
Sarawak clinical *B. pseudomallei* isolates kill rate (%) at the different sulfamethoxazole concentrations (µg/mL) after 18 h in broth. Isolates with the suffixes (b) and (c) represent subcultures or derivatives of the primary strain, denoted by suffix (a). For example, SWK-C 149 (b) and (c) were derived from SWK-C 149 (a). This naming convention was consistently applied to all isolates subcultured from their respective primary strains. The arrows indicate the bacterial kill rates at 50% and 80% whereas the boxes indicate the modes of MIC readings at MIC_50_ and MIC_80_.

**Figure 5:**
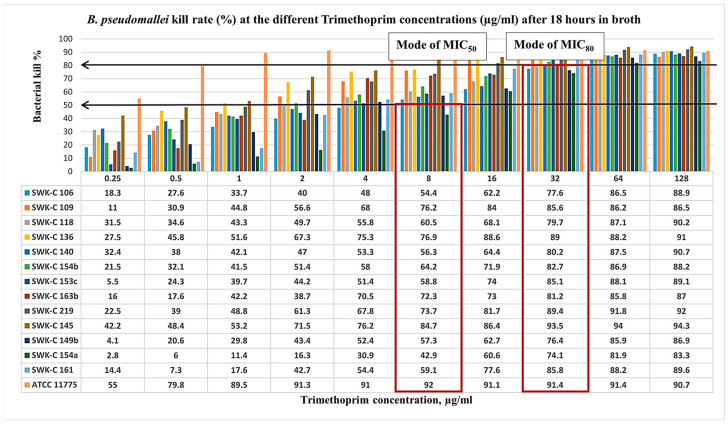
Sarawak clinical *B. pseudomallei* isolates kill rate (%) at the different trimethoprim concentrations (µg/mL) after 18 h in broth. Isolates with the suffixes (b) and (c) represent subcultures or derivatives of the primary strain, denoted by suffix (a). For example, SWK-C 149 (b) and (c) were derived from SWK-C 149 (a). This naming convention was consistently applied to all isolates subcultured from their respective primary strains. The arrows indicate the bacterial kill rates at 50% and 80% whereas the boxes indicate the modes of MIC readings at MIC50 and MIC80.

Following 18 h of broth microdilution testing with serial concentrations of sulfamethoxazole (SMX) and trimethoprim (TMP), the epidemiological MIC values of the isolates were analyzed (Figure 4 and Figure 5). Interestingly, the findings revealed that resistance to individual agents (SMX or TMP) did not confer resistance to the combined drug, trimethoprim-sulfamethoxazole. While all isolates were susceptible to SMX at MIC_50_ 4 to 32 μg/mL, half of the isolates (7/14) were resistant at MIC_80_ (Figure 4). Among these, four isolates exhibited low-level resistance of MIC_80_, ranging from 512 to 1024 μg/mL, and three isolates demonstrated high-level resistance of MIC_80_ more than 1024 µg/mL. Susceptible isolates were inhibited at MIC_80_ values of 32 to 256 µg/mL, with a mode of 128 µg/mL. A similar trend was observed for TMP (Figure 5). All isolates were susceptible at MIC_50_ ranging from 1 to 8 μg/mL, but the majority (13/14) were resistant at MIC_80_ ranging from 16 to 128 μg/mL, with the modal MIC_80_ of 32 μg/mL.

In Table 10, a comparison of the MIC values reveals that 7 trimethoprim-sulfamethoxazole-susceptible isolates were, in fact, resistant to both SMX and TMP at MIC_80_. Intriguingly, these isolates exhibited SMX MIC_80_ values higher than those of the trimethoprim-sulfamethoxazole-intermediate isolate SWK-C 118, and all were gentamicin-susceptible. Despite resistance to the individual agents, the synergistic combination of trimethoprim-sulfamethoxazole (co-trimoxazole) remained potent against all isolates, as confirmed by the E-test, with a predominant MIC of 2 µg/mL.

### 3.4. PCR Analysis Suggests Divergence in the bpeEF-oprC Efflux Pump Gene Cluster in Sarawak B. pseudomallei Clinical Isolates

To investigate the molecular basis of the observed sulfamethoxazole resistance and its impact on the trimethoprim-sulfamethoxazole resistance, we targeted the *bpeEF-oprC* efflux pump gene cluster, a known determinant of trimethoprim-sulfamethoxazole resistance in *B. pseudomallei*. Thirteen primer sets were designed based on the alignment of reference strain *B. pseudomallei* K96243 (accession: BX571996.1) with sequences from closely related Thai and Malaysian isolates to ensure broad applicability.

**Figure 6:**
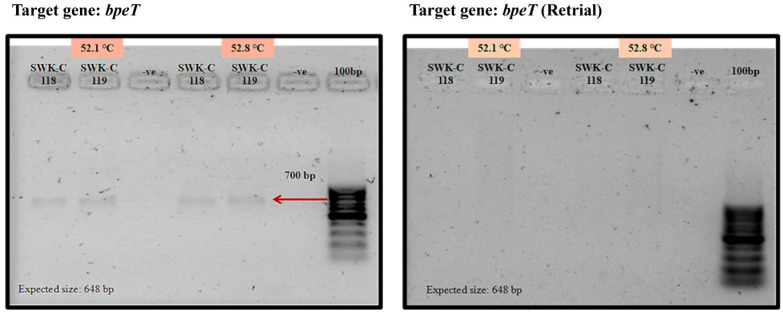
Analysis of the amplified *bpeT* from Sarawak *B. pseudomallei* clinical isolates.

Despite extensive optimization efforts, including gradient PCR, we were unable to successfully amplify the *bpeEF-oprC* gene cluster (*bpeT-llpE-bpeE-bpeF-oprC*) from any of the Sarawak clinical isolates. Although a faint product (Figure 6) was initially observed for the *bpeT* gene, this result was not reproducible in subsequent trials.

## 4. Discussion

Sarawak, Malaysian Borneo, is endemic for a unique gentamicin-susceptible (Gen^S^) population of *B. pseudomallei* belonging to the multilocus sequence type (ST)881 and its single-locus variant ST997 [12,13]. Comprehensive antibiotic susceptibility data for Sarawak clinical isolates remain scarce. Apart from the established Gen^S^ prevalence [12,13], only a single pediatric case of rare ceftazidime resistance has been reported [23]. Notably, some isolates from prior studies [12,13] were included in the present analysis (denoted in Appendix A Table A1 and Table A2).

This study demonstrates that the Gen^S^ genotype maintains high susceptibility to trimethoprim-sulfamethoxazole (SXT), reaffirming its clinical utility for melioidosis eradication therapy in this region. Our results indicated an estimated susceptibility prevalence of 96.3% (CLSI) and 97.6% (EUCAST). As confirmatory E-testing was limited to a subset of intermediate or resistant isolates, the estimated prevalence should be interpreted with caution. Despite that, this estimated prevalence aligns with the prevalence rates across Southeast Asia and Australia [24,25,26]. It is also consistent with a previous broader study of Malaysian isolates (which included 33/170 from Sarawak), where 90% of the isolates were SXT-susceptible [27]. The sustained in vitro susceptibility suggests SXT retains its bactericidal efficacy in Sarawak, which also corresponds to stable clinical treatment outcomes. Therefore, the findings of our present study resolve the concerns from previous reports of elevated MICs in Sarawak [10,11]. Our findings also recontextualize those reports to reflect methodological challenges in ASTs rather than a genuine loss or reduction in the clinical efficacy of SXT.

Another critical finding of our work is that disk diffusion testing significantly overcalled SXT resistance, a phenomenon previously documented in other endemic regions [22,23]. This overestimation resulted from visual ambiguity in some IZ phenotypes, particularly those with unclear or diffuse zone edges irrespective of haze growth (Figure 2). In addition, reader subjectivity is another major variable which impacts MIC estimation and the final susceptibility call [28]. This methodological challenge was attributed to the absence of a standardized, pathogen-specific AST methodology for *B. pseudomallei* at the time of the initiation of this study. The gap necessitated our local laboratories to adapt guidelines for *Enterobacteriaceae* and other *non-Enterobacteriaceae*. To ensure accurate reporting despite these constraints, we implemented a two-step AST algorithm by verifying non-susceptible disk diffusion results with an E-test. Our study offers a practical solution for AST reporting in cases where IZ cannot be confidently determined. Where resources permit, we recommend integrating a two-step AST into a standardized melioidosis diagnostic framework for Sarawak.

Our analysis further validated the CLSI breakpoints as the more reliable interpretive standard for these Sarawak isolates. While both 2017 CLSI and 2018 EUCAST criteria confirmed high susceptibility, EUCAST breakpoints yielded a substantially higher reported resistance rate (16.5% versus 2.4% by CLSI, Table 7). This higher rate defined by EUCAST, due in part to its more stringent susceptible breakpoint, could unnecessarily discourage the use of trimethoprim-sulfamethoxazole therapy.

Notably, our investigation coincided with EUCAST establishing its first standardized methodology for *B. pseudomallei* AST [28]. This new framework directly addresses our identified challenges, providing clear guidance for interpreting hazy or double inhibition zones via 80% growth inhibition measurement [28]. It also revises the ambiguous “intermediate” category to “susceptible, increased exposure” [29,30]. This update should reassure clinicians and bolster confidence without altering treatment regimens [9]. Therefore, while our data are aligned well with the CLSI standard, we recommend the gradual adoption of the pathogen-specific 2020 EUCAST guideline for future AST and surveillance works of *B. pseudomallei* in Sarawak. This will align local practices with the global standard, enhancing the consistency and clinical relevance of AST reporting.

Intriguingly, we found that the high in vitro efficacy of SXT persists despite intrinsic resistance to its individual components. Broth microdilution revealed that a substantial subset of SXT-susceptible isolates exhibited resistance to both SMX and TMP when assessed individually at MIC_80_. This finding aligns with observations from other endemic regions (northeast Thailand and northern Australia), where TMP resistance alone is common and does not compromise SXT efficacy [21]. In *B. pseudomallei*, such resistance is frequently attributed to the overexpression of the BpeEF-oprC efflux pump rather than target modification, indicating efflux as the predominant resistance mechanism [21]. The occurrence of SMX resistance, however, is rare. To our knowledge, this may be the first report of such resistance in Gen^S^ *B. pseudomallei* clinical isolates. In fact, the functional basis of SMX resistance in this pathogen remains largely uncharacterized. A previous study [31] suggested that BpeEF-OprC expression due to *bpeT* mutations or pump overexpression due to *bpeS* mutations, which is responsible for the TMP resistance, contributed to the reduced SMX susceptibility in regulatory mutants.

Additionally, this phenomenon occurred in isolates that otherwise maintained full susceptibility to clinically relevant antibiotics, including meropenem, ceftazidime, doxycycline, and amoxicillin-clavulanate. This suggests that their resistance to TMP and SMX is a specific anomaly, not part of a multidrug-resistant phenotype. This paradox underscores the paramount importance of drug synergy, whereby the combined formulation overcomes resistance for either drug alone. Hence, the unexpected resistance to the individual agents does not diminish the clinical utility of trimethoprim-sulfamethoxazole, which remains a cornerstone of melioidosis eradication therapy in Sarawak.

A key limitation of this study, however, is our inability to define the genetic basis for this phenotype. We were unable to amplify the *bpeEF-oprC* efflux pump gene cluster using primers designed against classic, gentamicin-resistant strains. The consistent failure to amplify the known resistance determinant [20] does not exclude the presence of an efflux-mediated resistance mechanism but rather suggests significant sequence divergence in this locus within the Gen^S^ Sarawak genotype. It is plausible that the unique lineage of ST881 and ST997 with distinguished mutation in the *amrB* efflux pump gene [12] also harbors distinct variations within the *bpeEF-oprC* operon. This is a compelling hypothesis that warrants future investigation through whole-genome sequencing.

In conclusion, our work provides strong evidence that SXT remains an effective eradication therapy for melioidosis in Sarawak. We recommend the use of CLSI guidelines, supplemented with the E-test for non-susceptible disk diffusion results, to ensure accurate AST reporting. The difference between individual and combination drug susceptibility, coupled with the genetic uniqueness of these isolates, highlights that the resistance mechanisms in this endemic population may be distinct. Future genomic studies utilizing next-generation sequencing of the Gen^S^ Sarawak *B. pseudomallei* clinical isolates are essential to elucidating these underlying mechanisms. These methods generate continuous sequence reads spanning the entire operons. This capability will allow for the comprehensive detection of structural variation or novel mutations within the complex resistance loci like the *bpeEF-oprC* efflux pump gene cluster, which could not be amplified with only PCR.

## Figures and Tables

**Table 1:** Number of Sarawak clinical *B. pseudomallei* isolates collected from the hospitals.

Hospital	Location	No. of Isolates Collected
Bintulu Hospital	Bintulu, Sarawak	98
Sibu Hospital	Sibu, Sarawak	10
Kapit Hospital	Kapit, Sarawak	45
Miri Hospital	Miri, Sarawak	7
Sarawak General Hospital	Kuching, Sarawak	2
Borneo Medical Centre	Kuching, Sarawak	2

**Table 2:** E-test MIC’s interpretation reference for the antibiotics tested in this study.

Antibiotic	MIC Interpretation According to Both CLSI and EUCAST Standards (µg/mL)		
	S≤	I	R≥
Trimethoprim-sulfamethoxazole (SXT)	2	-	4
Amoxicillin-clavulanate (AMC)	8/4	16/8	32/16
Azithromycin (AZ)	2	4	8
Ceftazidime (TZ)	8	16	32
Doxycycline (DC)	4	8	16
Gentamicin (GEN)	4	8	16
Meropenem (MEM)	2	8	2

Extracted from Document M100, 27th Edition, CLSI [14] and EUCAST [15] guidelines Version 8.1.

**Table 3:** MIC values of the tested isolates and *E. coli* ATCC 11775 against trimethoprim-sulfamethoxazole based on disk diffusion and the E-test method.

Isolates	Description	Zone Diameter, mm			MIC, µg/mL		
		Trial 1	Trial 2	Trial 3	Trial 1	Trial 2	Trial 3
SWK-C 106	SXT^I^ in this study ^1^	12	16	14	2	3	2
SWK-C 118	SXT^I^ in this study ^1^	12	16	12	3	2	2
SWK-C 145	SXT^S^ in this study ^2^	20	22	22	0.75	0.75	0.5
*E. coli* ATCC 25922	QC recommended by CLSI and EUCAST	NZ ^3^	NZ ^3^	NZ ^3^	NZ ^3^	NZ ^3^	NZ ^3^
*E. coli* ATCC 11775	Alternative QC proposed in this study	32	32	33	0.125	0.125	0.125

^1^ SXT^I^ = Trimethoprim-sulfamethoxazole-intermediate isolate. ^2^ SXT^S^ = Trimethoprim-sulfamethoxazole-susceptible isolate. ^3^ NZ = No inhibition zone.

**Table 4:** MIC values of the tested isolates and *E. coli* ATCC 11775 against trimethoprim-sulfamethoxazole with prolonged incubation.

Isolates	Zone Diameter (mm)			MIC (µg/mL)		
	18 h	24 h	36 h	18 h	24 h	36 h
SWK-C 106	16	11	NZ	3	4	NZ
SWK-C 118	16	16	9	2	4	NZ
SWK-C 145	22	16	13	0.75	1.5	3
*E. coli* ATCC 25922	NZ	NZ	NZ	NZ	NZ	NZ
*E. coli* ATCC 11775	32	32	32	0.125	0.125	0.125

NZ = No inhibition zone.

**Table 5:** Oligonucleotide primers designed and used in this study.

Target Gene	Primer	Primer Sequence (5′->3′) [Expected Base Pair, bp]
*bpeT*	BpeT_F1 BpeT_R1 BpeT_F2 BpeT_R2	5′- TGC GCA AAC ATA TGA CGA AC -3′ 5′- CGA ATT CCA CTC ACG CTA CC -3′ [768 bp] 5′- GCG GCT CGA AAA GTA GTT GA -3′ 5′- ACA ATT CAC GTC CCC TGA AC -3′ [684 bp]
*llpe*	llpE_F1 llpE_R1 llpE_F2 llpE_R2	5′- GAT TGT TCA GGG GAC GTG A -3′ 5′- GAG CGA ATA ATC GAC CGA CA -3′ [392 bp] 5′- CGG TGG TGC TTT ATT TCC AC -3’ 5′- CGG GAA GTA CGC AAG ATA GC -3′ [774 bp]
*bpeE*	BpeE_F1 BpeE_R1 BpeE_F2 BpeE_R2	5′- CGA CAA CCT GAG GGG TTT T -3′ 5′- GCC GAT GTA TTG CAG GTA GG -3′ [730 bp] 5′- TTA CGA CGA GAA GCA GAA CG -3′ 5′- TGA AAG GCT CTG TCT GAT TGG -3′ [846 bp]
*bpeF*	BpeF_F1 BpeF_R1 BpeF_F2 BpeF_R2 BpeF_F3 BpeF_R3 BpeF_F4 BpeF_R4 BpeF_F5 BpeF_R5	5′- CCC AAT CAG ACA GAG CCT TT -3′ 5′- CGA ACT CGT CCT CGT TCT G -3′ [769 bp] 5′- ATT CGC GAG CAG AAC GTG -3′ 5′- GTC ATC GCG AAC TGC TTG TA -3′ [797 bp] 5′- CTA TTC GAT CAA CGC GCT CT -3′ 5′- CCG CGT ACT TCT GGT TCA G -3′ [824 bp] 5′- GTG AAC GGC TTC ACG AAC A -3′ 5′- TGA TCG GAA ACA CCC AGA AC -3′ [796 bp] 5′- GAC ATC CTG CAA CTG AAG ACG -3’ 5′- GCG TTC GTT GAT GTT GGT CT -3′ [850 bp]
*oprC*	OprC_F1 OprC_R1 OprC_F2 OprC_R2	5′- CGG ACG CTT GAG GAT AGA AA -3′ 5′- CTC GCT GAA CGA GAA ATC C -3′ [882 bp] 5′- CGC GGA TTT CTC GTT CAG -3′ 5′- CGA CAT TCG CAT TTC GTC -3′ [785 bp]

**Table 6:** Trimethoprim-sulfamethoxazole (25 µg disk) inhibition zone diameter distributions for the Sarawak clinical *B. pseudomallei* isolates.

IZD ^1^ (mm)	≤10	11	12	13	14	15	16	17	18	19	20	21	22	23	24	25	26	27	28	29	≥30
MIC_80_, 1 IZ ^2^	2	1	7	1	2	10	6	2	5	11	**15** ^5^	**15** ^5^	7	2	6	6	4	0	2	2	11
MIC_50_, 2 IZs ^3^	0	0	0	0	0	0	0	0	0	0	0	0	1	1	2	3	3	4	**6** ^5^	4	23
MIC_80_, 2 IZs ^4^	1	0	2	0	0	4	3	3	4	3	**10** ^5^	4	6	1	4	1	1	0	0	0	0
Total, N = 164 (MIC_80_)	3	1	9	1	2	14	9	5	9	14	**25** ^5^	19	13	3	10	7	5	0	2	2	11

^1^ IZD is the abbreviation for the inhibition zone diameter (mm). ^2^ MIC_80_, 1 IZ refers to the inhibition zone diameter of n = 117 isolates with one (1) inhibition zone. ^3^ MIC_50_, 2 IZs refers to the innermost zone margin of n = 47 isolates with two (2) inhibition zones. ^4^ MIC_80_, 2 IZs refers to the outermost zone margin of n = 47 isolates with two (2) inhibition zones. ^5^ The bold and underlined number is the mode for the inhibition zone diameter (mm).

**Table 7:** Comparisons of CLSI and EUCAST susceptibility interpretations for the Sarawak clinical *B. pseudomallei* isolates against trimethoprim-sulfamethoxazole (25 µg disk).

MIC’s Interpretive Standard	CLSI Interpretive Standard		EUCAST Interpretive Standard	
	Frequency, % (n/N)	95% CI	Frequency, % (n/N)	95% CI
Susceptible Isolates	81.7% (134/164)	0.749 ~ 0.873	79.9% (131/164)	0.729 ~ 0.857
Intermediate Isolates	15.9% (26/164)	0.106 ~ 0.224	3.7% (6/164)	0.014 ~ 0.078
Resistant Isolates	2.4% (4/164)	0.007 ~ 0.061	16.5% (27/164)	0.111 ~ 0.230
Cohen’s Kappa value, κ (95% CI)	κ = 0.458 (0.340 ~ 0.570)			

Cohen’s Kappa value (κ) interpretation of the prevalence variability [16]: κ = 0.00–0.20, indicates slight agreement; κ = 0.21–0.40, indicates fair agreement; κ = 0.41–0.60, indicates moderate agreement; κ = 0.61–0.80, indicates substantial agreement; κ = 0.81–1.00 indicates almost perfect agreement.

**Table 8:** Mathematically corrected susceptibility of trimethoprim-sulfamethoxazole among the Sarawak clinical *B. pseudomallei* isolates.

Frequency (%)	CLSI’s Standard		EUCAST’s Standard	
	Disk Diffusion	E-Test	Disk Diffusion	E-Test
Susceptibility, % (n/N)	81.7% (134/164)	96.3% (158/164)	79.9% (131/164)	97.6% (160/164)
Intermediate, % (n/N)	15.9% (26/164)	3.7% (6/164)	3.7% (6/164)	1.2% (2/164)
Resistance, % (n/N)	2.4% (4/164)	-	16.5% (27/164)	1.2% (2/164)

**Table 9:** Antibiotic susceptibility profile of 14 isolates selected for broth microdilution.

SXT Phenotype (Disk Diffusion)	Isolate(s)	SXT	GEN	AZ	MEM	TZ	DC	AMC
**Susceptible**	**SWK-C 145**	0.38 (S)	NZ (R)	NZ (R)	1.5 (S)	1.5 (S)	1 (S)	3 (S)
	**SWK-C 133**	0.5 (S)	1 (S)	3 (S)	1 (S)	2 (S)	0.75 (S)	4 (S)
	**SWK-C 136**	0.5 (S)	2 (S)	4 (S)	0.75 (S)	1.5 (S)	0.75 (S)	3 (S)
	**SWK-C 161**	0.5 (S)	2 (S)	4 (S)	2 (S)	2 (S)	0.75 (S)	3 (S)
**Resistance**	**SWK-C 219**	0.5 (S)	96 (R)	NZ (R)	2 (S)	3 (S)	1.5 (S)	4 (S)
	**SWK-C 109**	1 (S)	1.5 (S)	6(S)	1 (S)	1.5 (S)	0.75 (S)	6 (S)
	**SWK-C 118**	3 (I)	6 (S)	6(S)	1.5 (S)	2 (S)	1 (S)	6 (S)
**Intermediate**	**SWK-C 106**	2 (S)	1.5 (S)	4 (S)	1 (S)	2 (S)	0.75 (S)	4 (S)
	**SWK-C 140**	2 (S)	6 (S)	4 (S)	1.5 (S)	2 (S)	0.75 (S)	4 (S)
	**SWK-C 149 (b)**	2 (S)	1.5 (S)	4 (S)	1.5 (S)	3 (S)	0.75 (S)	6 (S)
	**SWK-C 154 (a)**	2 (S)	1.5 (S)	3 (S)	1.5 (S)	2 (S)	0.75 (S)	4 (S)
	**SWK-C 154 (b)**	2 (S)	1.5 (S)	4 (S)	2 (S)	2 (S)	0.75 (S)	6 (S)
	**SWK-C 154 (c)**	2 (S)	4 (S)	4 (S)	2 (S)	1.5 (S)	0.75 (S)	4 (S)
	**SWK-C 163 (b)**	2 (S)	2 (S)	4 (S)	1.5 (S)	3 (S)	1 (S)	4 (S)
**E-test Susceptibility frequency (%)**		92.9%	85.7%	85.7%	100%	100%	100%	100%

Isolates with the suffixes (b) and (c) represent subcultures or derivatives of the primary strain, denoted by suffix (a). For example, SWK-C 149 (b) and (c) were derived from SWK-C 149 (a). This naming convention was consistently applied to all isolates subcultured from their respective primary strains. Abbreviations: SXT = Trimethoprim-sulfamethoxazole; GEN = Gentamicin; MEM = Meropenem; TZ = Ceftazidime; DC = Doxycycline; AZ = Azithromycin; AMC = Amoxicillin-clavulanate; I = Intermediate; R = Resistance; S = Susceptible; NZ = No inhibition zone.

**Table 10:** Comparisons of the MICs and susceptibility interpretation for 14 Sarawak clinical *B. pseudomallei* isolates.

Isolate(s)	Disk Diffusion IZD (mm)	E-Test MIC (µg/mL)	SMX, Broth Microdilution MIC (µg/mL)		TMP, Broth Microdilution MIC (µg/mL)	
			MIC_50_	MIC_80_	MIC_50_	MIC_80_
SWK-C 219 ^GenR^	9 (R)	2 (S)	8 (S)	128 (S)	2 (S)	16 (R)
SWK-C 163b ^GenS^	10 (R)	2 (S)	32 (S)	**1024 (R)** ^1^	4 (S)	**32 (R)** ^1^
SWK-C 106 ^GenS^	12 (I)	2 (S)	32 (S)	**512 (R)** ^1^	8 (S)	**64 (R)** ^1^
SWK-C 118 ^GenS^	12 (I)	3 (I)	8 (S)	256 (S)	4 (S)	64 (R)
SWK-C 154b ^GenS^	12 (I)	2 (S)	8 (S)	128 (S)	2 (S)	32 (R)
SWK-C 140 ^GenS^	15 (I)	2 (S)	16 (S)	**1024 (R)** ^1^	4 (S)	**32 (R)** ^1^
SWK-C 154c ^GenS^	14 (I)	2 (S)	16 (S)	**512 (R)** ^1^	4 (S)	**32 (R)** ^1^
SWK-C 149b ^GenS^	16 (S)	2 (S)	16 (S)	256 (S)	4 (S)	64 (R)
SWK-C 154a ^GenS^	16 (S)	2 (S)	32 (S)	**ETR** ^1,2^	16 (R)	**64 (R)** ^1^
SWK-C 109 ^GenS^	18 (S)	1 (S)	32 (S)	**ETR** ^1,2^	2 (S)	**16 (R)** ^1^
SWK-C 145 ^GenR^	20 (S)	0.75 (S)	4 (S)	32 (S)	1 (S)	8 (S)
SWK-C 133 ^GenS^	21 (S)	0.75 (S)	4 (S)	128 (S)	2 (S)	16 (R)
SWK-C 136 ^GenS^	21 (S)	0.25 (S)	4 (S)	32 (S)	1 (S)	16 (R)
SWK-C 161 ^GenS^	37 (S)	0.5 (S)	32 (S)	**ETR** ^1,2^	4 (S)	**32 (R)** ^1^

Isolates with the suffixes (b) and (c) represent subcultures or derivatives of the primary strain, denoted by suffix (a). For example, SWK-C 149 (b) and (c) were derived from SWK-C 149 (a). This naming convention was consistently applied to all isolates subcultured from their respective primary strains. Abbreviations: S = Susceptible; R = Resistance; and I = Intermediate. ^1^ Bold and underlined details referred to MIC cut-offs of the isolate that are resistant to both SMX and TMP. ^2^ ETR indicates that the MIC exceeded the concentration range tested.

## Data Availability

Further information and data are available from the corresponding author upon reasonable request.

## Abbreviations

The following abbreviations are used in this manuscript:

MIC	Minimal inhibitory concentration
CLSI	Clinical Laboratory Standards Institute
EUCAST	European Committee on Antimicrobial Susceptibility Testing
QC	Quality Control
IZ	Inhibition zone
AST	Antimicrobial Susceptibility Testing
MIC_50_	Minimal inhibitory concentration at 50% inhibited growth
MIC_80_	Minimal inhibitory concentration at 80% inhibited growth
OD	Optical density
Gen^S^	Gentamicin-susceptible
TMP	Trimethoprim
SMX	Sulfamethoxazole
PCR	Polymerase Chain Reaction
ST	Sequence type

## Appendix A

Table A1 in this section showed the overall disk diffusion results of the trimethoprim-sulfamethoxazole susceptibility of the Sarawak isolates, detailed with the source location. Some of the isolates were previously studied in [12], characterizing the Gentamicin susceptibility profile (see Table A1). The bolded isolates in Table A1 then selected for the E-test against trimethoprim-sulfamethoxazole, which results were tabulated in Table A2.

**Table A1:** Disk diffusion results of 164 Sarawak clinical *B. pseudomallei* isolates when tested against the 25 µg Trimethoprim-sulfamethoxazole disk.

Isolates	Origin	MIC50, mm (for Type C IZ Only)	MIC_80_, mm (CLSI/EUCAST Interpretation)	Type of Inhibition Zone
SWK-C 101	Bintulu	NA	19 (S/S)	
**SWK-C 103** ^1**,3**^	Bintulu	NA	10 (R/R)	**Type B** ^1^
SWK-C 105	Bintulu	NA	23 (S/S)	
**SWK-C 106** ^1**,3**^	Bintulu	NA	12 (I/R)	**Type B** ^1^
SWK-C 107	Bintulu	NA	25 (S/S)	
**SWK-C 108** ^1**,3**^	Bintulu	NA	11 (I/R)	**Type B** ^1^
**SWK-C 109** ^1**,3**^	Bintulu	NA	18 (S/R)	**Type B** ^1^
SWK-C 111	Bintulu	NA	18 (S/S)	
SWK-C 114	Bintulu	NA	26 (S/S)	
SWK-C 115	Bintulu	NA	21 (S/S)	
**SWK-C 118 ^1^**	Bintulu	NA	12 (I/R)	**Type B** ^1^
**SWK-C 119 ^1^**	Bintulu	NA	15 (I/R)	**Type B** ^1^
SWK-C 120	Bintulu	NA	34 (S/S)	
**SWK-C 123** ^1**,3**^	Bintulu	NA	14 (I/R)	**Type B** ^1^
SWK-C 143	Bintulu	NA	24 (S/S)	
**SWK-C 145** ^1^	Bintulu	NA	20 (S/S)	**Type B** ^1^
SWK-C 146 (a)	Bintulu	NA	19 (S/S)	
SWK-C 146 (b)	Bintulu	NA	21 (S/S)	
**SWK-C 146 (c)** ^1**,3**^	Bintulu	NA	15 (I/R)	**Type B** ^1^
SWK-C 147	Bintulu	NA	19 (S/S)	
SWK-C 148	Bintulu	NA	19 (S/S)	
SWK-C 149 (a)	Bintulu	NA	18 (S/S)	
**SWK-C 149 (b)** ^1^	Bintulu	NA	16 (S/R)	**Type B** ^1^
**SWK-C 150** ^1**,3**^	Bintulu	NA	15 (I/R)	**Type B** ^1^
SWK-C 151	Bintulu	NA	20 (S/S)	
**SWK-C 154 (b)** ^1**,3**^	Bintulu	NA	12 (I/I)	**Type B** ^1^
**SWK-C 154 (c)** ^1^	Bintulu	NA	14 (I/R)	**Type B** ^1^
**SWK-C 155 (a)** ^1^	Bintulu	NA	12 (I/R)	**Type B** ^1^
**SWK-C 155 (b)** ^1^	Bintulu	NA	18 (S/R)	**Type B** ^1^
SWK-C 156	Bintulu	NA	20 (S/S)	
**SWK-C 158** ^1**,3**^	Bintulu	NA	12 (I/R)	**Type B** ^1^
SWK-C 159	Bintulu	NA	17 (S/S)	
SWK-C 160	Bintulu	NA	37 (S/S)	
**SWK-C 161** ^1^	Bintulu	NA	37 (S/R)	**Type B** ^1^
SWK-C 162	Bintulu	NA	25 (S/S)	
**SWK-C 163 (a)** ^1**,3**^	Bintulu	NA	15 (I/R)	**Type B** ^1^
**SWK-C 163 (b)** ^1**,3**^	Bintulu	NA	10 (R/R)	**Type B** ^1^
SWK-C 164	Bintulu	NA	20 (S/S)	
SWK-C 167	Bintulu	NA	21 (S/S)	
SWK-C 172	Bintulu	NA	21 (S/S)	
SWK-C 175	Bintulu	NA	21 (S/S)	
SWK-C 177 **^3^**	Bintulu	NA	15 (I/R)	
SWK-C 180	Bintulu	NA	25 (S/S)	
SWK-C 182	Bintulu	NA	23 (S/S)	
SWK-C 184	Bintulu	NA	24 (S/S)	
SWK-C 185	Bintulu	NA	24 (S/S)	
MSHR 7905	Bintulu	NA	22 (S/S)	
MSHR 7895	Bintulu	NA	28 (S/S)	
MSHR 7903	Bintulu	NA	21 (S/S)	
MSHR 7881	Bintulu	NA	17 (S/S)	
MSHR 7882	Bintulu	NA	20 (S/S)	
MSHR 7906	Bintulu	NA	20 (S/S)	
MSHR 7887 **^3^**	Bintulu	NA	22 (S/S)	
MSHR 7888	Bintulu	NA	25 (S/S)	
SWK-C 192	Bintulu	NA	20 (S/S)	
SWK-C 193	Bintulu	NA	30 (S/S)	
**SWK-C 194** ^1^	Bintulu	NA	15 (I/R)	**Type B** ^1^
SWK-C 195	Bintulu	NA	21 (S/S)	
SWK-C 196	Bintulu	NA	20 (S/S)	
SWK-C 197	Bintulu	NA	28 (S/S)	
**SWK-C 199** ^1^	Bintulu	NA	16 (S/R)	**Type B** ^1^
SWK-C 243	Bintulu	NA	16 (S/S)	
SWK-C 267	Bintulu	NA	12 (I/I)	
SWK-C 269	Bintulu	NA	13 (I/I)	
SWK-C 270	Bintulu	NA	20 (S/S)	
SWK-C 271	Bintulu	NA	16 (S/S)	
SWK-C 290	Bintulu	NA	15 (I/S)	
SWK-C 124	Kapit	NA	19 (S/S)	
SWK-C 125	Kapit	NA	24 (S/S)	
SWK-C 126	Kapit	NA	22 (S/S)	
SWK-C 127	Kapit	NA	19 (S/S)	
SWK-C 130	Kapit	NA	22 (S/S)	
**SWK-C 131** ^1^	Kapit	NA	19 (S/S)	**Type A** ^1^
**SWK-C 132** ^1^	Kapit	NA	20 (S/S)	**Type A** ^1^
**SWK-C 133** ^1^	Kapit	NA	21 (S/S)	**Type A** ^1^
**SWK-C 134** ^1^	Kapit	NA	19 (S/S)	**Type A** ^1^
**SWK-C 136** ^1^	Kapit	NA	21 (S/S)	**Type A** ^1^
**SWK-C 138** ^1^	Kapit	NA	16 (S/S)	**Type A** ^1^
**SWK-C 139** ^1^	Kapit	NA	15 (R/S)	**Type B** ^1^
**SWK-C 140** ^1^	Kapit	NA	15 (I/R)	**Type B** ^1^
SWK-C 141	Kapit	NA	24 (S/S)	
SWK-C 214	Kapit	NA	22 (S/S)	
SWK-C 215	Kapit	NA	24 (S/S)	
SWK-C 216	Kapit	NA	22 (S/S)	
SWK-C 218	Kapit	NA	19 (S/S)	
SWK-C 220	Kapit	NA	20 (S/S)	
**SWK-C 221** ^1^	Kapit	NA	20 (S/R)	**Type B** ^1^
SWK-C 222	Kapit	NA	19 (S/S)	
**SWK-C 223** ^1^	Kapit	NA	12 (I/I)	**Type B** ^1^
SWK-C 226	Kapit	NA	16 (S/S)	
SWK-C 227	Kapit	NA	26 (S/S)	
SWK-C 229	Kapit	NA	30 (S/S)	
SWK-C 230	Kapit	NA	26 (S/S)	
SWK-C 231	Kapit	NA	19 (S/S)	
**SWK-C 233** ^1^	Kapit	NA	**15 (I/R)** ^1^	**Type B** ^1^
SWK-C 234	Kapit	NA	20 (S/S)	
SWK-C 235	Kapit	NA	30 (S/S)	
SWK-C 236	Kapit	NA	19 (S/S)	
SWK-C 237	Kapit	NA	29 (S/S)	
SWK-C 238	Kapit	NA	21 (S/S)	
SWK-C 241	Kapit	NA	21 (S/S)	
SWK-C 242	Kapit	NA	33 (S/S)	
SWK-C 089	Kapit	NA	21 (S/S)	
SWK-C 097	Kuching	NA	20 (S/S)	
SWK-C 187	Kuching	NA	20 (S/S)	
MSHR 7891	Miri	NA	21 (S/S)	
MSHR 7894	Miri	NA	25 (S/S)	
SWK-C 063 **^3^**	Sibu	NA	30 (S/S)	
SWK-C 064 **^3^**	Sibu	NA	20.7 (S/S)	
MSHR 6392 ^2^	Sibu	NA	31 (S/S)	
MSHR 6802 ^2^	Sibu	NA	29 (S/S)	
MSHR 6401 ^2^	Sibu	NA	25 (S/S)	
MSHR 6404 ^2^	Sibu	NA	18 (S/S)	
MSHR 6816 ^2^	Sibu	NA	31 (S/S)	
SWK-C 084	Sibu	NA	22 (S/S)	
SWK-C 085	Sibu	NA	26 (S/S)	
SWK-C 087	Sibu	NA	21 (S/S)	
SWK-C 102	Bintulu	35	21 (S/S)	
SWK-C 104	Bintulu	32	22 (S/S)	
**SWK-C 112** ^1^	Bintulu	23	**15 (I/S)** ^1^	**Type C** ^1^
**SWK-C 113** ^1**,3**^	Bintulu	22	**12 (I/I)** ^1^	**Type C** ^1^
SWK-C 116	Bintulu	35	21 (S/S)	
SWK-C 117	Bintulu	28	20 (S/S)	
SWK-C 152	Bintulu	30	16 (S/S)	
SWK-C 153	Bintulu	33	24 (S/S)	
**SWK-C 154 (a)** ^1^	Bintulu	24	**16 (S/S)** ^1^	**Type C** ^1^
SWK-C 157	Bintulu	34	20 (S/S)	
SWK-C 165	Bintulu	29	20 (S/S)	
SWK-C 168	Bintulu	28	18(S/S)	
SWK-C 169	Bintulu	29	20 (S/S)	
SWK-C 170	Bintulu	25	19 (S/S)	
SWK-C 173	Bintulu	27	17 (S/S)	
SWK-C 176	Bintulu	30	18 (S/S)	
MSHR 7883	Bintulu	29	24 (S/S)	
MSHR 7884	Bintulu	27	22 (S/S)	
MSHR 7896	Bintulu	35	20 (S/S)	
MSHR 7904	Bintulu	32	17 (S/S)	
MSHR 7885	Bintulu	35	26 (S/S)	
**MSHR 7897** ^1^	Bintulu	32	**15 (I/S)** ^1^	**Type C** ^1^
MSHR 7886	Bintulu	29	18 (S/S)	
SWK-C 200	Bintulu	30	20 (S/S)	
SWK-C 201	Bintulu	24	17 (S/S)	
SWK-C 204	Bintulu	27	20 (S/S)	
SWK-C 207	Bintulu	31	21 (S/S)	
SWK-C 209	Bintulu	25	20 (S/S)	
SWK-C 210	Bintulu	32	25 (S/S)	
SWK-C 212	Bintulu	33	22 (S/S)	
SWK-C 262 **^3^**	Bintulu	26	16 (S/S)	
**SWK-C 129** ^1^	Kapit	26	**15 (I/S)** ^1^	**Type C** ^1^
SWK-C 213	Kapit	33	22 (S/S)	
**SWK-C 219** ^1^	Kapit	28	**9 (R/R)** ^1^	**Type C** ^1^
SWK-C 224	Kapit	28	22 (S/S)	
SWK-C 225	Kapit	35	24 (S/S)	
SWK-C 228	Kapit	25	23 (S/S)	
**SWK-C 232** ^1^	Kapit	33	**12 (I/I)** ^1^	**Type C** ^1^
**SWK-C 239** ^1^	Kapit	33	**15 (I/S)** ^1^	**Type C** ^1^
SWK-C 240	Kapit	32	24 (S/S)	
**SWK-C 096** ^1^	Kuching	28	**18 (S/S)** ^1^	**Type C** ^1^
SWK-C 100	Kuching	30	20 (S/S)	
MSHR 7898/M2	Miri	27	21 (S/S)	
MSHR 7899/M3	Miri	31	22 (S/S)	
MSHR 7889/M4	Miri	26	19 (S/S)	
MSHR 7890/M5	Miri	28	19 (S/S)	
MSHR 7892/M7	Miri	30	20 (S/S)	

Isolates with the suffixes (b) and (c) represent subcultures or derivatives of the primary strain, denoted by suffix (a). For example, SWK-C 149 (b) and (c) were derived from SWK-C 149 (a). This naming convention was consistently applied to all isolates subcultured from their respective primary strains. Abbreviations: S = Susceptible; I = Intermediate; R = Resistance; and NA = Not applicable. ^1^ The bold details refer to the isolate and the criteria that were selected for the E-test. ^2^ Isolates previously studied in [12]. ^3^ Isolates involved in an unpublished study characterizing Ceftazidime and Meropenem susceptibility.

**Table A2:** Minimum inhibitory concentration results of Sarawak clinical *B. pseudomallei* isolates against the trimethoprim-sulfamethoxazole E-test strip.

Isolate(s)	Origin	Minimal Inhibitory Concentration ((µg/mL)	MIC Interpretation (CLSI and EUCAST)
SWK-C 103 ^2^	Bintulu	1	S
SWK-C 106 ^2^	Bintulu	2	S
SWK-C 108 ^2^	Bintulu	0.75	S
SWK-C 109 ^2^	Bintulu	1	S
SWK-C 112	Bintulu	0.5	S
SWK-C 113 ^2^	Bintulu	0.75	S
**SWK-C 118**	**Bintulu**	**3** **^1^**	**I**
SWK-C 119	Bintulu	1.5	S
SWK-C 123 ^2^	Bintulu	1	S
SWK-C 145	Bintulu	0.38	S
SWK-C 146 (c) ^2^	Bintulu	1.5	S
SWK-C 149 (b)	Bintulu	2	S
SWK-C 150 ^2^	Bintulu	1.5	S
SWK-C 154 (a)	Bintulu	2	S
SWK-C 154 (b) ^2^	Bintulu	2	S
SWK-C 154 (c)	Bintulu	2	S
SWK-C 155 (a)	Bintulu	1.5	S
SWK-C 155 (b)	Bintulu	1.5	S
SWK-C 158	Bintulu	1	S
SWK-C 161	Bintulu	0.5	S
SWK-C 163 (a) ^2^	Bintulu	1.5	S
SWK-C 163 (b) ^2^	Bintulu	2	S
MSHR 7897	Bintulu	2	S
SWK-C 194	Bintulu	0.38	S
SWK-C 199	Bintulu	0.5	S
SWK-C 129	Kapit	0.75	S
SWK-C 131	Kapit	1	S
SWK-C 132	Kapit	1	S
SWK-C 133	Kapit	0.75	S
SWK-C 134	Kapit	0.5	S
SWK-C 136	Kapit	1.5	S
SWK-C 138	Kapit	0.5	S
SWK-C 139	Kapit	0.75	S
SWK-C 140	Kapit	1	S
SWK-C 219	Kapit	2	S
SWK-C 221	Kapit	0.5	S
SWK-C 223	Kapit	0.75	S
SWK-C 226	Kapit	1.5	S
SWK-C 232	Kapit	1	S
SWK-C 233	Kapit	0.75	S
SWK-C 239	Kapit	0.75	S
SWK-C 096	Kuching	0.75	S

Isolates with the suffixes (b) and (c) represent subcultures or derivatives of the primary strain, denoted by suffix (a). For example, SWK-C 149 (b) and (c) were derived from SWK-C 149 (a). This naming convention was consistently applied to all isolates subcultured from their respective primary strains. Abbreviations: S = Susceptible; and I = Intermediate. ^1^ Bold and underlined number refers to the intermediate MIC (µg/mL). ^2^ Isolates involved in an unpublished study characterizing Ceftazidime and Meropenem susceptibility.

## References

[1] Currie B.J., Kaestli M. (2016). A global picture of melioidosis. Nature.

[2] Limmathurotsakul D., Golding N., Dance D.A.B., Messina J.P., Pigott D.M., Moyes C.L., Rolim D.B., Bertherat E., Day N.P.J., Peacock S.J. (2016). Predicted global distribution of *Burkholderia pseudomallei* and burden of melioidosis. Nat. Microbiol..

[3] Wiersinga W.J., Virk H.S., Torres A.G., Currie B.J., Peacock S.J., Dance D.A.B., Limmathurotsakul D. (2018). Melioidosis. Nat. Rev. Dis. Primers.

[4] Sanchez-Villamil J.I., Torres A.G. (2018). Melioidosis in Mexico, Central America, and the Caribbean. Trop. Med. Infect. Dis..

[5] Nathan S., Chieng S., Kingsley P.V., Mohan A., Podin Y., Ooi M.-H., Mariappan V., Vellasamy K.M., Vadivelu J., Daim S. (2018). Melioidosis in Malaysia: Incidence, Clinical Challenges, and Advances in Understanding Pathogenesis. Trop. Med. Infect. Dis..

[6] Sia T.L.L., Mohan A., Ooi M.-H., Chien S.-L., Tan L.-S., Goh C., Pang D.C.L., Currie B.J., Wong J.-S., Podin Y. (2021). Epidemiological and Clinical Characteristics of Melioidosis Caused by Gentamicin-Susceptible *Burkholderia pseudomallei* in Sarawak, Malaysia. Open Forum Infect. Dis..

[7] Mohan A., Podin Y., Tai N., Chieng C.-H., Rigas V., Machunter B., Mayo M., Wong D., Chien S.-L., Tan L.-S. (2017). Pediatric melioidosis in Sarawak, Malaysia: Epidemiological, clinical and microbiological characteristics. PLoS Neglected Trop. Dis..

[8] White N. (2003). Melioidosis. Lancet.

[9] Dance D. (2014). Treatment and prophylaxis of melioidosis. Int. J. Antimicrob. Agents.

[10] Vima V., Ling H.-W., Cho W.-M., Norhuzaimah J. (2016). Incidence, Risk Factors and Clinical Epidemiology of Melioidosis in Miri Hospital, Sarawak, Malaysia. Sarawak Health J..

[11] Yong K.-Y., Tang A.S.-O., Teh Y.-C., Fam T.-L., Chua H.-H. (2016). Prevalence of Antibiotic Resistance in *Burkholderia pseudomallei* Cases Presented to Miri General Hospital. Sarawak Health J..

[12] Podin Y., Kaestli M., McMahon N., Hennessy J., Ngian H.U., Wong J.S., Mohana A., Wong S.C., William T., Mayo M. (2013). Reliability of Automated Biochemical Identification of Burkholderia pseudomallei Is Regionally Dependent. J. Clin. Microbiol..

[13] Podin Y., Sarovich D.S., Price E.P., Kaestli M., Mayo M., Hii K., Ngian H., Wong S., Wong I., Wong J. (2013). Burkholderia pseudomallei Isolates from Sarawak, Malaysian Borneo, Are Predominantly Susceptible to Aminoglycosides and Macrolides. Antimicrob. Agents Chemother..

[14] CLSI Performance Standards for Antimicrobial Susceptibility Testing 27th ed. CLSI Supplement M100.

[15] European Committee on Antimicrobial Susceptibility Testing (2018). Breakpoint Tables for Interpretation of MICs and Zone Diameters. Version 8.1, Valid from 2018-05-15. https://www.eucast.org/ast_of_bacteria/previous_versions_of_documents/.

[16] CLSI (2018). Methods for Dilution Antimicrobial Susceptibility Tests for Bacteria That Grow Aerobically 11th ed CLSI Standard M07.

[17] Warrens M.J. (2015). Five Ways to Look at Cohen’s Kappa. J. Psychol. Psychother..

[18] Untergasser A., Cutcutache I., Koressaar T., Ye J., Faircloth B.C., Remm M., Rozen S.G. (2012). Primer3—New capabilities and interfaces. Nucleic Acids Res..

[19] Holden M.T.G., Titball R.W., Peacock S.J., Cerdeño-Tárraga A.M., Atkins T., Crossman L.C., Pitt T., Churcher C., Mungall K., Bentley S.D. (2004). Genomic plasticity of the causative agent of melioidosis, *Burkholderia pseudomallei*. Proc. Natl. Acad. Sci. USA.

[20] Biot F.V., Valade E., Garnotel E., Chevalier J., Villard C., Thibault F.M., Vidal D.R., Pagès J.-M. (2011). Involvement of the Efflux Pumps in Chloramphenicol Selected Strains of Burkholderia thailandensis: Proteomic and Mechanistic Evidence. PLoS ONE.

[21] Podnecky N.L., Wuthiekanun V., Peacock S.J., Schweizer H.P. (2013). The BpeEF-OprC Efflux Pump Is Responsible for Widespread Trimethoprim Resistance in Clinical and Environmental *Burkholderia pseudomallei* Isolates. Antimicrob. Agents Chemother..

[22] Payne G.W., Vandamme P., Morgan S.H., LiPuma J.J., Coenye T., Weightman A.J., Jones T.H., Mahenthiralingam E. (2005). Development of a *recA* Gene-Based Identification Approach for the Entire *Burkholderia* Genus. Appl. Environ. Microbiol..

[23] Sia T.L.-L., Lai C.D., Manan K., Khiu F.-L., Bakhtiar S.Z., Chor Y.-K., Chien S.-L., Tan L.-S., Ooi M.-H., Mohan A. (2025). Ceftazidime-resistance in pediatric melioidosis: A case report and literature review. IDCases.

[24] Dance D., Davong V., Soeng S., Phetsouvanh R., Newton P., Turner P. (2014). Trimethoprim/sulfamethoxazole resistance in Burkholderia pseudomallei. Int. J. Antimicrob. Agents.

[25] Saiprom N., Amornchai P., Wuthiekanun V., Day N.P., Limmathurotsakul D., Peacock S.J., Chantratita N. (2015). Trimethoprim/sulfamethoxazole resistance in clinical isolates of Burkholderia pseudomallei from Thailand. Int. J. Antimicrob. Agents.

[26] Dutta S., Haq S., Hasan M.R., Haq J.A. (2017). Antimicrobial susceptibility pattern of clinical isolates of Burkholderia pseudomallei in Bangladesh. BMC Res. Notes.

[27] Ahmad N., Hashim R., Noor A.M. (2013). The In Vitro Antibiotic Susceptibility of Malaysian Isolates of *Burkholderia pseudomallei*. Int. J. Microbiol..

[28] Karatuna O., Dance D., Matuschek E., Åhman J., Turner P., Hopkins J., Amornchai P., Wuthiekanun V., Cusack T.-P., Baird R. (2021). Burkholderia pseudomallei multi-centre study to establish EUCAST MIC and zone diameter distributions and epidemiological cut-off values. Clin. Microbiol. Infect..

[29] Dance D.A., Wuthiekanun V., Baird R.W., Norton R., Limmathurotsakul D., Currie B.J. (2021). Interpreting Burkholderia pseudomallei disc diffusion susceptibility test results by the EUCAST method. Clin. Microbiol. Infect..

[30] Kahlmeter G., Giske C.G., Kirn T.J., Sharp S.E. (2019). Point-Counterpoint: Differences between the European Committee on Antimicrobial Susceptibility Testing and Clinical and Laboratory Standards Institute Recommendations for Reporting Antimicrobial Susceptibility Results. J. Clin. Microbiol..

[31] Podnecky N.L., Rhodes K.A., Mima T., Drew H.R., Chirakul S., Wuthiekanun V., Schupp J.M., Sarovich D.S., Currie B.J., Keim P. (2017). Mechanisms of Resistance to Folate Pathway Inhibitors in *Burkholderia pseudomallei*: Deviation from the Norm. mBio.

